# Supplementary material for: Profiling the lymphoid-resident T cell pool reveals modulation by age and microbiota
Source: Nat Commun. 2018 Jan 4;9:68. doi: 10.1038/s41467-017-02458-4 (PMC5754350; doi:10.1038/s41467-017-02458-4)
Supplement: Supplementary file 1 — Supplementary Information [file 41467_2017_2458_MOESM1_ESM.pdf]

SUPPLEMENTARY INFORMATION

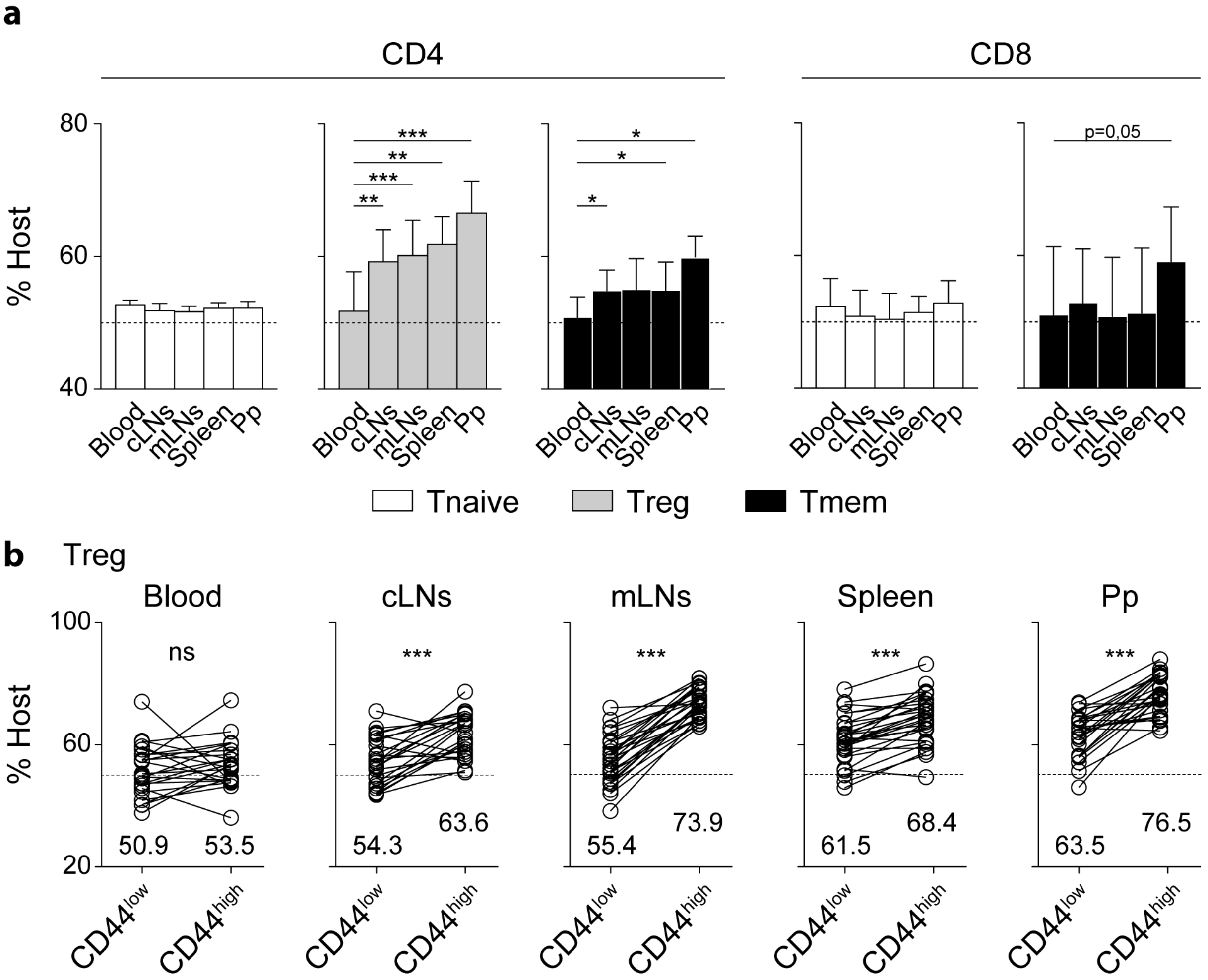

Supplementary Figure 1: Analysis of mouse parabionts reveals the long-term residence of regulatory and memory T cell populations in SLOs. (**a**) Eight weeks after parabiosis surgery, blood, cervical LNs (cLNs), mesenteric LNs (mLNs), spleen and Peyer's patches (Pp) were recovered and analyzed. Proportions of host cells ($CD45.1^+$ for the CD45.1 parabiont and $CD45.2^+$ for the CD45.2 parabiont) among the indicated CD4 T cell and CD8 T cell subsets recovered from blood, cLNs, mLNs, Spleen and Pp are shown as means ± SEM. (**b**) Four weeks after parabiosis surgery, proportions of host cells among $CD44^{low}$ and $CD44^{high}$ CD4 Treg cells. Each pair of dots represents an individual mouse. Significant differences are indicated by asterisks (paired t-test, *p < 0.05, **p < 0.01, ***p < 0.001).

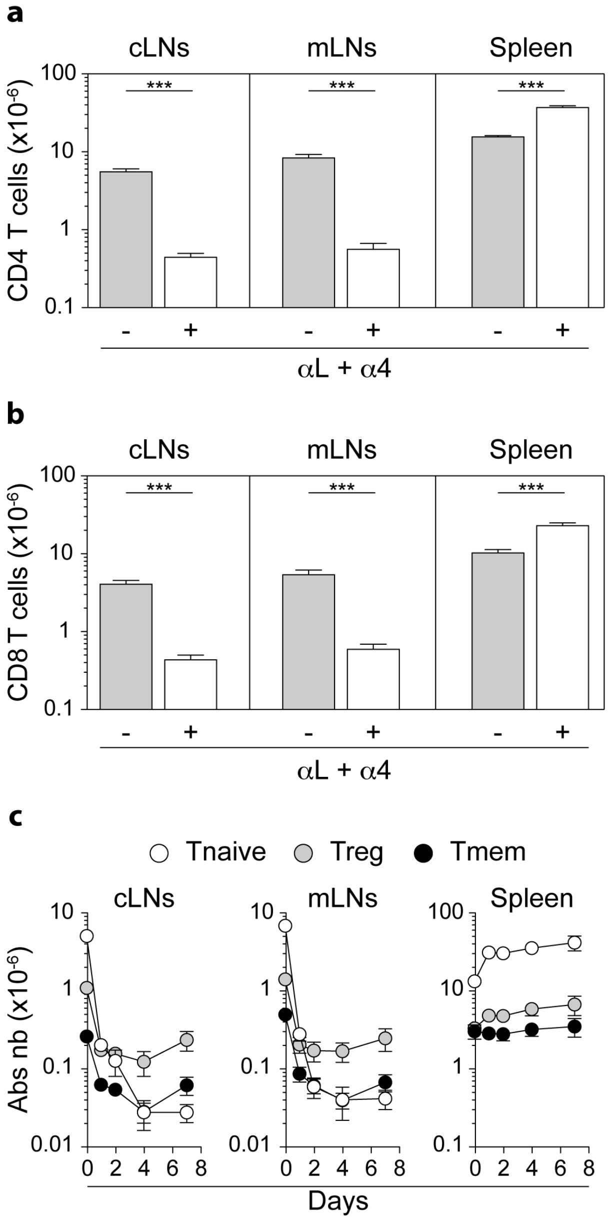

Supplementary Figure 2: Analysis of residual T cells after blocking T cell entry into LNs. 6-12 week-old C57BL/6 Foxp3-GFP mice were injected or not i.p. with 200 μg of anti-LFA-1 (αL) and anti-VLA-4 (α4) Abs. Twenty-four hours later, cLNs, mLNs and spleen were harvested and analyzed. (**a**) Absolute numbers of CD4 T cells in the indicated SLOs of treated or untreated mice are shown as means ± SEM with unpaired *t*-test or at least three independent experiments (**b**) Same than in (**a**) for CD8 T cells. (**c**) C57BL/6 Foxp3-GFP mice were injected or not i.p. with 200 μg of anti-LFA-1 (αL) and anti-VLA-4 (α4) Abs every 2 days from day 0 to day 6 and SLOs were recovered for analysis at various time-points. Absolute numbers of CD4 Treg, Tmem and Tnaive cells in cLNs, mLNs, and spleen are shown. *p < 0.05, **p < 0.01, ***p < 0.001. ns, not significant.

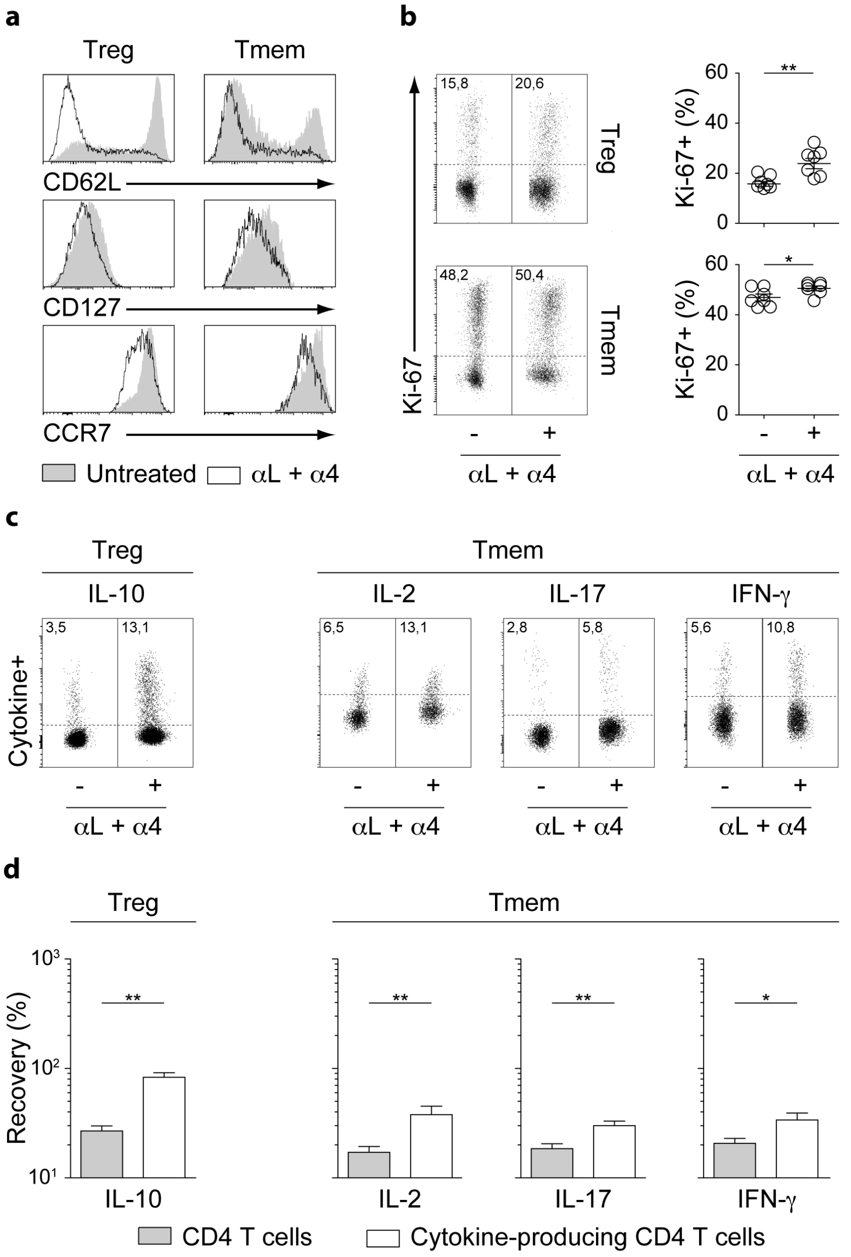

Supplementary Figure 3: mLN-resident CD4 T cells exhibit an effector phenotype. 6-12 week-old C57BL/6 Foxp3-GFP mice were injected or not i.p. with 200 µg of anti-LFA-1 (αL) and anti-VLA-4 (α4) Abs. Forty-eight hours later, mLNs were harvested and analyzed. (**a**) CD62L, CD127 and CCR7 fluorescence histograms of CD4 Treg and CD4 Tmem cells from a representative treated and a representative control C57BL/6 Foxp3-GFP mouse. (**b**) Representative Ki-67 expression by CD4 Treg and CD4 Tmem cells from treated and control mice. Quantification is shown on the right part of the panel. Each dot represents an individual mouse (unpaired *t*-test). (**c**) Representative IL-10 expression by CD4 Treg cells and representative IL-2, IL-17 and IFN-γ expression by CD4 Tmem cells from treated and control mice. (**d**) Quantification of cytokine production by CD4 Treg and Tmem cells is shown as means ± SEM with paired *t*-test. The percentage of recovery for a given cytokine was calculated by dividing the absolute numbers of cells expressing this cytokine in treated mice by the mean absolute number of cells expressing this same cytokine in untreated animals (white bars). The grey bars correspond to the recovery of total CD4 Treg or CD4 Tmem cells in the same samples. *$p < 0.05$, **$p < 0.01$, ***$p < 0.001$. ns, not significant.

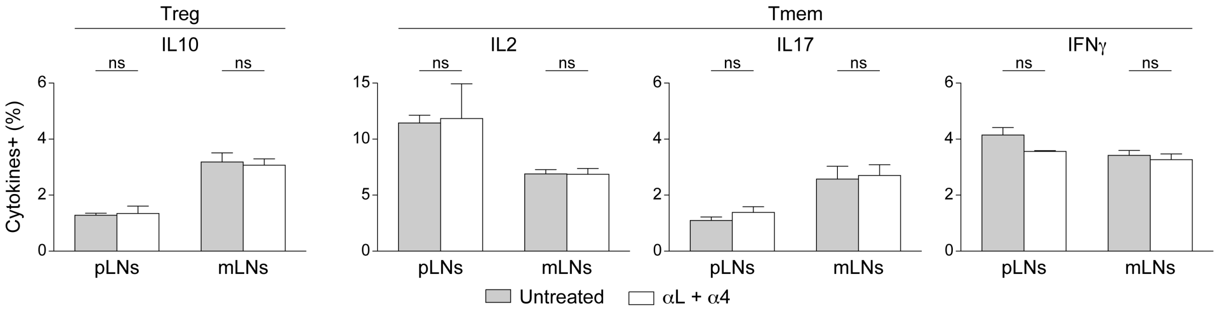

Supplementary Figure 4: Integrin engagement does not potentiate T cell ability to produce cytokines. pLNs and mLNs from 6-12 week-old C57BL/6 Foxp3-GFP mice were harvested. Cells from pLNs and mLNs were preincubated or not with anti-LFA-1 (αL) and anti-VLA-4 (α4) Abs (50 μg/ml) for 30 minutes at 4°C. They were then stimulated with 0.5 mg/ml PMA, 0.5 mg/ml ionomycin, and 10 mg/ml brefeldin A (all from Sigma-Aldrich) for 2 h at 37°C. Cells were then stained for surface markers, fixed in 2% paraformaldehyde in PBS, and permeabilized with 0.5% saponin, followed by labeling with specific cytokine Abs. The percentage of CD4 Treg cells producing IL-10 and the percentages of CD4 Tmem cells producing IL-2, IL-17 or IFN-γ were determined and are shown as means ± SEM with paired *t*-test.

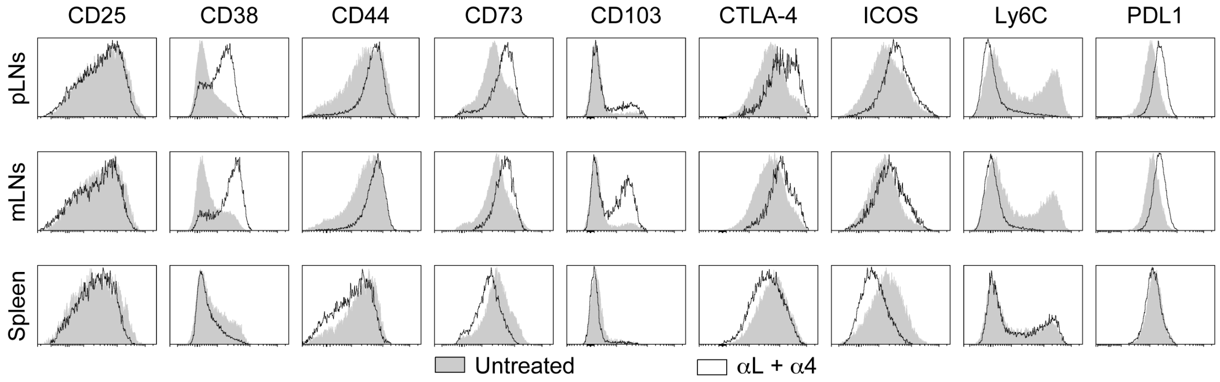

Supplementary Figure 5: Analysis of the LN-resident CD4 Treg cell phenotype. 6-12 week-old C57BL/6 Foxp3-GFP mice were injected or not i.p. with 200 μg of anti-LFA-1 (αL) and anti-VLA-4 (α4) Abs. Forty-eight hours later, pLNs, mLNs and spleen were harvested and analyzed. CD25, CD38, CD44, CD73, CD103, CTLA-4, ICOS, Ly-6C and PDL1 fluorescence histograms of CD4 Treg cells from a representative treated and a representative control C57BL/6 Foxp3-GFP mouse.

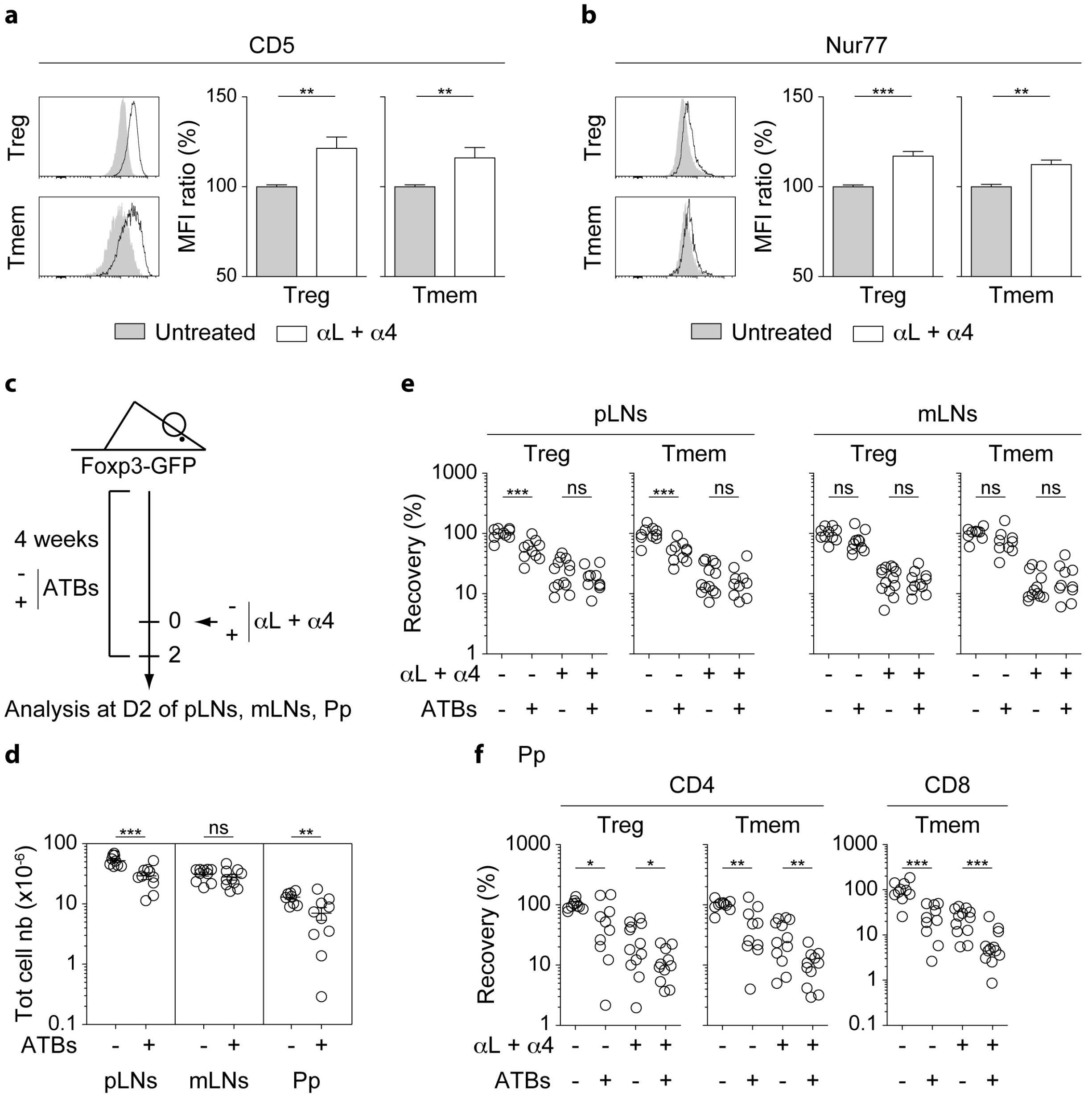

Supplementary Figure 6: Assessing the relative role of self-antigens and microbiota in T cell residence in SLOs. (**a-b**) 6-12 week-old C57BL/6 Foxp3-GFP mice were injected or not i.p. with 200 μg of anti-LFA-1 (αL) and anti-VLA-4 (α4) Abs. Forty-eight hours later, mesenteric LNs (mLN) were harvested and analyzed. CD5 (**a**) and Nur77 (**b**) fluorescence histograms of CD4 Treg and CD4 Tmem cells from the mLNs a representative treated and a representative control C57BL/6 Foxp3-GFP mouse. Quantification is shown as means ± SEM with unpaired *t*-test on the right part of these panels. (**c-f**) 6-8 week-old C57BL/6 Foxp3-GFP mice were treated with antibiotics (ATB) for 4 weeks. Then, ATB treated or untreated mice were injected or not i.p. with 200 μg of anti-LFA-1 (αL) and anti-VLA-4 (α4) Abs. Forty-eight hours later, SLOs were harvested and analyzed. (**c**) Diagram illustrating the experimental model. (**d**) Total cell numbers recovered from pLNs, mLNs and Pp of antibiotic treated or untreated mice (**e**) Recovery of CD4 Treg and CD4 Tmem cells in pLNs and mLNs. (**f**) Recovery of CD4 Treg, CD4 Tmem and CD8 Tmem cells in Pp. Each dot represents an individual mouse (unpaired *t*-test). $^*p < 0.05$, $^{**}p < 0.01$, $^{***}p < 0.001$. ns, not significant. Mouse clip arts were generated in (21).

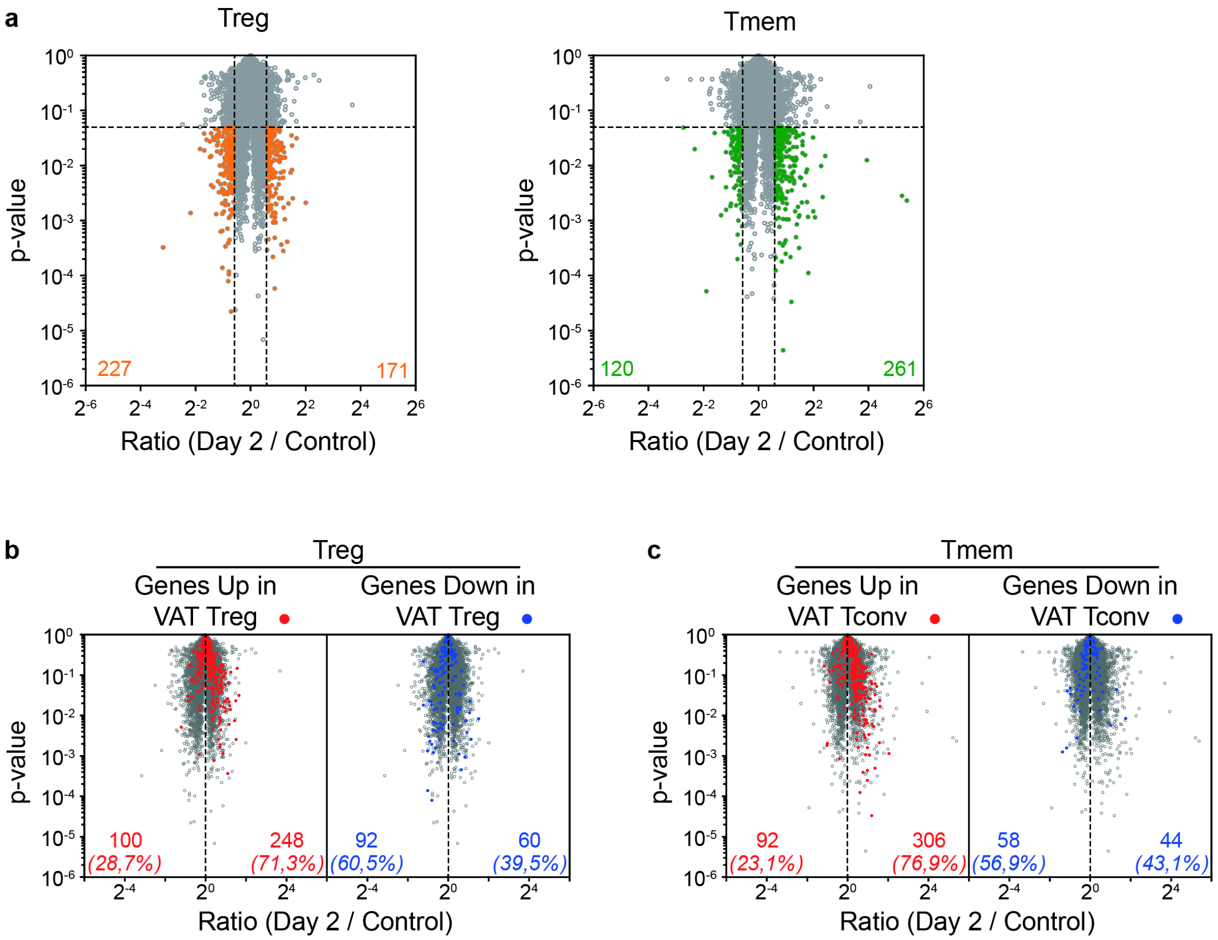

Supplementary Figure 7: Transcriptional profiling of LN- and VAT-resident CD4 Treg and Tmem cells. (**a**) "Volcano plot" representation (Log_2 (ratio) versus Log_{10} (t test p value)) between day 2 and control LN CD4 Treg (left) or CD4 Tmem (right) cells from C57BL/6 Foxp3-GFP mice. Genes expressed >1.5-fold higher or lower in day 2 LN CD4 cells compared to control LN CD4 cells with a *P* value of <0.05 are highlighted in orange (CD4 Treg cells) or green (CD4 Tmem cells). The number of genes up- or down-regulated (1.5-fold cutoff) for each comparison is indicated. (**b**) Among the 500 genes the most differentially expressed between VAT and control CD4 Treg cells with a *P* value of <0.05, up-regulated and down-regulated genes are highlighted in red and blue respectively. Datasets were filtered to common probes between the 2 arrays. (**c**) Same than in (**b**) for CD4 Tmem cells.
